# Anger and Optimism Moderate the Relationship Between Neighborhood Disadvantage and Biological Aging in MESA

**DOI:** 10.1093/geroni/igaf122.3706

**Published:** 2025-12-31

**Authors:** Joyce Woo, James Pike, Timothy Hughes, Jana Hirsch, Priya Palta, Ganga Bey

**Affiliations:** The University of North Carolina at Chapel Hill, Chapel Hill, North Carolina, United States; The University of North Carolina at Chapel Hill, Chapel Hill, North Carolina, United States; Atrium Health Wake Forest Baptist, Winston-Salem, North Carolina, United States; Drexel University, Philadelphia, Pennsylvania, United States; UNC School of Medicine, Chapel Hill, North Carolina, United States; The University of North Carolina at Chapel Hill, Chapel Hill, North Carolina, United States

## Abstract

We investigated whether individual-level psychosocial risk and resilience differentially moderate the relationship between neighborhood disadvantage and biological aging across ethnoracial groups. Using the Multi-Ethnic Study of Atherosclerosis (MESA), our study included 1,229 participants with DNA methylation (DNAm) data collected in 2010-2012. Neighborhood disadvantage proximal to DNAm assesment was captured using the 2015 Area Deprivation Index (ADI) at the census tract-level. Psychosocial factors included optimism, measured at Exam 2 (2002-2004) using the Life Orientation Test-Revised, and anger reactivity/temperament, measured at Exam 1 (2000-2002) using the State-Trait Anger Expression Inventory. Pace of aging was assessed as the difference between chronological age and GrimAge, a DNAm-based epigenetic clock. Mixed effects models with interaction terms evaluated whether the gender-adjusted relationship between ADI and pace of aging was moderated by optimism and anger. Among participants (mean age: 69.6 years, 51% female, 47% white, 21% Black, 31% Hispanic), greater neighborhood disadvantage was associated with increased age acceleration (β = 0.27, 95% CI: 0.05, 0.49). Unexpectedly, optimism amplified this association (β = 0.33, 95% CI: 0.05, 0.60). Stratified models revealed that moderation by optimism was present only for Hispanic participants (β = 1.73, 95% CI: 0.71, 2.74), whereas anger reactivity was predictive of slower biological aging (β = -1.63, 95% CI: -3.03, -0.24) among Black participants only. This study highlights the importance of modifiable psychosocial factors in shaping the impact of neighborhood disadvantage on aging. Our findings point to a need for interventions tailored to the unique sociocultural contexts of ethnoracial groups to mitigate disparities in aging.

